# BH3 mimetics and azacitidine show synergistic effects on juvenile myelomonocytic leukemia

**DOI:** 10.1038/s41375-023-02079-5

**Published:** 2023-11-09

**Authors:** Ying Wu, Patricia M. A. Zehnle, Jovana Rajak, Naile Koleci, Geoffroy Andrieux, Lorena Gallego-Villar, Konrad Aumann, Melanie Boerries, Charlotte M. Niemeyer, Christian Flotho, Sheila Bohler, Miriam Erlacher

**Affiliations:** 1 Department of Pediatrics and Adolescent Medicine, Division of Pediatric Hematology and Oncology, University Medical Center Freiburg, Faculty of Medicine, University of Freiburg, Freiburg, Germany; 2 Department of Biliary-Pancreatic Surgery, Renji Hospital Affiliated to Shanghai Jiao Tong University School of Medicine, Shanghai, China; 3 Department of Pediatrics and Adolescent Medicine, Division of General Pediatrics, University Medical Center Freiburg, Faculty of Medicine, University of Freiburg, Freiburg, Germany; 4 Spemann Graduate School of Biology and Medicine (SGBM), University of Freiburg, Freiburg, Germany; 5 Institute of Medical Bioinformatics and Systems Medicine, Medical Center—University of Freiburg, Faculty of Medicine, University of Freiburg, Freiburg, Germany; 6 University Medical Center Freiburg, Institute of Surgical Pathology, Faculty of Medicine, University of Freiburg, Freiburg, Germany; 7 German Cancer Consortium (DKTK), Partner Site Freiburg; and German Cancer Research Center (DKFZ), Heidelberg, Germany; 8 Present Address: Shanghai Cancer Institute, State Key Laboratory of Oncogenes and Related Genes, Shanghai, China

**Keywords:** Myeloproliferative disease, Myeloproliferative disease

## Abstract

Juvenile myelomonocytic leukemia (JMML) is an aggressive hematopoietic disorder of infancy and early childhood driven by constitutively active RAS signaling and characterized by abnormal proliferation of the granulocytic-monocytic blood cell lineage. Most JMML patients require hematopoietic stem cell transplantation for cure, but the risk of relapse is high for some JMML subtypes. Azacitidine was shown to effectively reduce leukemic burden in a subset of JMML patients. However, variable response rates to azacitidine and the risk of drug resistance highlight the need for novel therapeutic approaches. Since RAS signaling is known to interfere with the intrinsic apoptosis pathway, we combined various BH3 mimetic drugs with azacitidine in our previously established patient-derived xenograft model. We demonstrate that JMML cells require both MCL-1 and BCL-X_L_ for survival, and that these proteins can be effectively targeted by azacitidine and BH3 mimetic combination treatment. In vivo azacitidine acts via downregulation of antiapoptotic MCL-1 and upregulation of proapoptotic BH3-only. The combination of azacitidine with BCL-X_L_ inhibition was superior to BCL-2 inhibition in eliminating JMML cells. Our findings emphasize the need to develop clinically applicable MCL-1 or BCL-X_L_ inhibitors in order to enable novel combination therapies in JMML refractory to standard therapy.

## Introduction

Juvenile myelomonocytic leukemia (JMML) is a rare and aggressive hematopoietic disorder of infancy and early childhood. Classified by the International Consensus Classification of Myeloid Neoplasms and Acute Leukemias as an overlap myeloproliferative/myelodysplastic neoplasia it is characterized by abnormal proliferation of the granulocytic-monocytic blood cell lineage [1, 2]. Constitutive activation of the RAS/MAPK signaling pathway by mutations in *KRAS* and *NRAS* or their regulators *PTPN11* (coding for SHP2), *NF1*, and *CBL* can be found in about 90% of JMML cases [3]. The heterogeneous clinical presentation is caused by leukemic infiltrations in bone marrow, liver, lung, intestines, skin, and typically in spleen [1]. Therapeutic strategies differ depending on the genetic subtype but mostly rely on allogeneic hematopoietic stem cell transplantation (HSCT) for a curative treatment option [4]. Based on data from three independent clinical cohorts, the DNA methylation status was established as a prognostic factor allowing risk stratification of JMML patients, with the poorest outcome in the high methylation subgroup [5]. Dysregulated epigenetic patterns contribute to JMML pathogenesis, but also provide targets for novel therapeutic approaches. Using a patient-derived xenograft (PDX) model of JMML closely reflecting human disease [6], we have previously shown that the hypomethylating agent azacitidine effectively reduced leukemia burden and, most importantly, efficiently depleted leukemia-initiating cells [7]. In line with our results in the murine model, the AZA-JMML-001 phase 2 clinical trial recently proved the safety and efficacy of azacitidine as a bridge to transplant in newly diagnosed JMML [8]. However, not all JMML patients respond equally to azacitidine, illustrating the need for novel combination therapies.

BH3 mimetics, a promising class of antineoplastic drugs, exert antitumor activity via inhibition of prosurvival BCL-2 proteins and consecutive induction of apoptosis [9]. The BCL-2 protein family plays an important role in regulating the intrinsic apoptosis pathway with both proapoptotic (BAX, BAK, BIM, BMF, PUMA, tBID, NOXA, BAD, BIK, and HRK) and antiapoptotic family members (BCL-2, BCL-X_L_, MCL-1, A1/BFL, BCL-W, and BCL-B). The appropriate balance between these proteins, and thus between cell death and survival, is crucial for physiological hematopoiesis, while deregulation is frequently found in hematological neoplasias [10]. The BCL-2 protein inhibitors navitoclax and venetoclax have shown robust anticancer efficacy in both hematological and non-hematological malignancies, the latter being the only FDA-approved BH3 mimetic drug to date [9].

Activation of the RAS/MAPK signaling pathway results in activation of antiapoptotic and deactivation of proapoptotic BCL-2 proteins, thereby enhancing cell survival [11]. We, therefore hypothesized that targeting prosurvival BCL-2 proteins via BH3 mimetics could be effective as part of a combination therapy in JMML. In this study, we tested the BH3 mimetic ABT737, an analog of navitoclax, as single agent and in combination with azacitidine. ABT737 was chosen because of its broad spectrum inhibiting BCL-2, BCL-X_L_, and BCL-W [12]. In complementary in vitro experiments we also used inhibitors specific for BCL-2, BCL-X_L_, or MCL-1. We show that the combination of ABT737 with azacitidine effectively targets JMML cells. Furthermore, we identified the BCL-2 homolog BCL-X_L_ as the major prosurvival molecule in JMML under azacitidine treatment in xenograft mice. Synergistic lethality of JMML cells was also found for the combination of selective BCL-X_L_ and MCL-1 inhibition.

## Materials and methods

### JMML cells

Human cells were collected after obtaining informed consent from parents or legal guardians and approval from institutional review committees. Samples from JMML patients were collected in the context of the European Working Group of MDS in Childhood (EWOG-MDS). Clinical characteristics of the patients are described in Online Supplementary Table 1. Single-cell suspensions obtained from mashed spleens were subjected to density gradient centrifugation (Ficoll) to separate and cryopreserve mononuclear cells (MNC). MNCs were depleted from CD3^+^ T cells according to a protocol published earlier (MACS immunobeads, Miltenyi; <0.15% remaining T cells) [6].

### Xenotransplantation

All experiments were performed after approval from the local ethics committee and in compliance with German law. *Rag2*^*−/−*^*γc*^*−/−*^ mice were kept under specific pathogen-free conditions [13]. Newborn mice were sub-lethally irradiated with 2.5 Gy. After 6 h, 1 × 10^6^ JMML cells were resuspended in 30 μl sterile PBS and injected into the liver of a mouse. Alternatively, 5-week old mice were irradiated with 3 Gy and transplanted intravenously with 5 × 10^6^ viable MNCs. Eight weeks after transplantation, mice were allocated to the treatment groups. Treatment consisted of a four-week therapy block of ABT737 (75 mg/kg/d or 50 mg/kg/d; Selleckchem), or ABT199 (100 mg/kg/d; MedChemExpress), azacitidine (3 mg/kg/d or 0.75 mg/kg/d; Sigma) for two cycles of 5 consecutive days every 2 weeks, vehicle (administered daily), or control (no treatment). Mice were sacrificed on day 29 of therapy or followed until they were critically sick. For analysis, single cell suspensions were obtained from bone marrow, spleen, and blood. Liver and lung were digested with collagenase D and DNase (Roche) followed by density gradient centrifugation. For serial transplantation, engrafted primary recipient mice were sacrificed 29 days after the start of therapy, and 10 × 10^6^ bone marrow cells were transplanted into sub-lethally irradiated secondary recipients at the age of 5 weeks. Successful engraftment was defined by the proportion of 0.5% or more human CD45^+^ cells in murine organs, following an accepted convention for xenografts [14, 15].

### Flow cytometry

Patient-derived JMML cells or mechanically-dissociated mouse tissue cells were surface stained with monoclonal antibodies from Biolegend and Becton Dickinson: CD45-mouse Horizon Blue V605 (30-F11), CD45-human PE (HI30), CD11b AlexaFluor488 (M1/70), CD38 PerCP/Cy5.5 (HIT2), CD34 PE/Cy7 (581), CD33 APC (WM53), CD13 APC/Cy7 (WM15), CD14 Pacific Blue (M5E2), CD19 FITC (HIB19), CD66b PerCP/Cy5.5 (G10F5), CD3 PE/Cy7 (SK7), CD71 APC/Cy7 (CY1G4), CD235a BV421 (GA-R2). For intracellular staining, live cells were first stained for surface proteins and then fixed, permeabilized, and stained for intracellular proteins using the Foxp3 Transcription Factor Staining Buffer Set (Invitrogen) according to the manufacturer’s instructions. For intracellular staining, the following antibodies were used: BCL-2 PE (3F11 BD), MCL-1 PE (Y37 Abcam), BCL-XL FITC (H-5 Santa Cruz) and matching isotype controls. Normalized median fluorescence intensity (MFI) was calculated according to the following equation: normalized MFI = (MFI of protein of interest) − (MFI of each isotype). Flow cytometry was performed using a BD LSRFortessa; for analyses FlowJo software was used. The gating strategy is shown in Online Supplementary Fig. S1.

### JMML cell culture

JMML spleen cell aliquots were thawed at 37 °C and subsequently resuspended in JMML medium (IMDM + 10% FCS + 1% P/S) to which 200 µl DNase (2 ng/ml) was added. In order to remove DMSO, the cells were rinsed twice with 25 ml JMML medium and centrifuged at 300 g for 10 min. Clotted material was dissolved by incubation in Accumax (eBioscience) at 37 °C for 20 min. The purified cells were then used for culture. 1 × 10^5^ mononuclear JMML spleen cells were resuspended in 100 μl IMDM medium +10% FCS + 1% P/S and mixed with cytokines including SCF and FLT3L at a concentration of 100 ng/ml, TPO at a concentration of 50 ng/ml, and IL-3 at a concentration of 10 ng/ml. Cells were cultured in a 96-well plate with U-shape bottom at 37 °C with 5% O_2_ and 5% CO_2_.

### Apoptosis assay in JMML cells

Apoptosis of JMML cells was assessed under combined treatment with BH3 mimetics and azacitidine. In 96-well plates, 10^5^ JMML MNCs per well were treated with BH3 mimetics (ABT737 (Selleckchem), S63845 (Synthesis), ABT199 (Selleckchem) or A1155463 (Selleckchem)), azacitidine (0.03, 0.1, 0.3, 1, or 3 µM), or a combination of both for 72 h. Concentrations of BH3 mimetics: ABT737: 0.1, 0.3, 1, 3, 10 µM; S63845: 0.03, 0.1, 0.3, 1, 3 µM; ABT199: 0.1, 0.3, 1, 3, 10 µM; A1155463: 0.1, 0.3, 1, 3, 10, 30 µM. After 72 h, the cells were stained with Annexin V-Alexa Fluor 647 (Biolegend) and 7-AAD (Sigma-Aldrich) and apoptosis was analyzed by flow cytometry. The same experiments were performed with human cord blood-derived CD34^+^ cells for comparison.

### Synergy scores

The degree of synergy or antagonism of two drugs was quantified using the following synergy scores: highest single agent (HSA), Bliss, Loewe, and Zero interaction potency model [16]. Briefly, the observed drug combination response was compared against the expected response in a reference model assuming no interaction between these drugs. A synergy score higher than 10 indicates a likely synergistic interaction between two drugs.

### Immunohistochemistry

Organs were fixed in 4% buffered formalin, and sternums were decalcified. After paraffin-embedding, sections were deparaffinized in xylene and graded alcohols. H&E staining followed standard protocols. Immunohistochemical staining was performed after specific antigen retrieval in “low pH target retrieval solution” (Dako) for 30 min. Primary antibodies CD34 (Agilent DAKO, Clone QBEnd 10, monoclonal mouse anti human, RTU) and CD45 (Agilent DAKO, Clone 2B11 + PD7/26, monoclonal mouse anti human, RTU) were used. The EnVision FLEX System or the APK5005 system were used for visualization (Dako). Sections were counterstained with hematoxylin (Dako) and mounted. Stainings without primary antibodies served as negative controls.

### RNA sequencing

RNA was isolated from indicated cells using the RNeasy micro kit (Qiagen) according to the manufacturer’s protocol. Isolated RNA was quantified (Qubit RNA assay kit, Thermo Fisher Scientific) and quality was assessed using an Agilent 2100 Bioanalyzer. RNA was further processed by the Genomics & Proteomics core facility of the German Cancer Research Center, Heidelberg, Germany, and sequenced using the Illumina HiSeq 4000 system with 100 bp paired-end sequencing. Adapter sequences and bad quality reads were trimmed using Trimmomatic (v0.38) [17] with the following parameters: HEADCROP:3 TRAILING:10 MINLEN:25. Reads were then aligned to the Human reference genome (hg19) and quantified using STAR (v2.5.2b) [18]. Differential analysis was performed with a linear model-based approach (limma R/Bioconductor package) using a paired design matrix [19]. Adjusted p-value (Benjamini Hochberg) below 0.05 was considered as significant.

### Statistical analysis

Bar graphs represent mean values and standard errors of the mean (SEM). The Mann-Whitney test was used to determine significance. Differences in survival (Kaplan-Meier curves) were analyzed by the Mantel–Cox log-rank test (GraphPad Prism 7.05). *P* < 0.05 was considered statistically significant.

## Results

### ABT737 and azacitidine both effectively target JMML cells in vivo

**Fig. 1:**
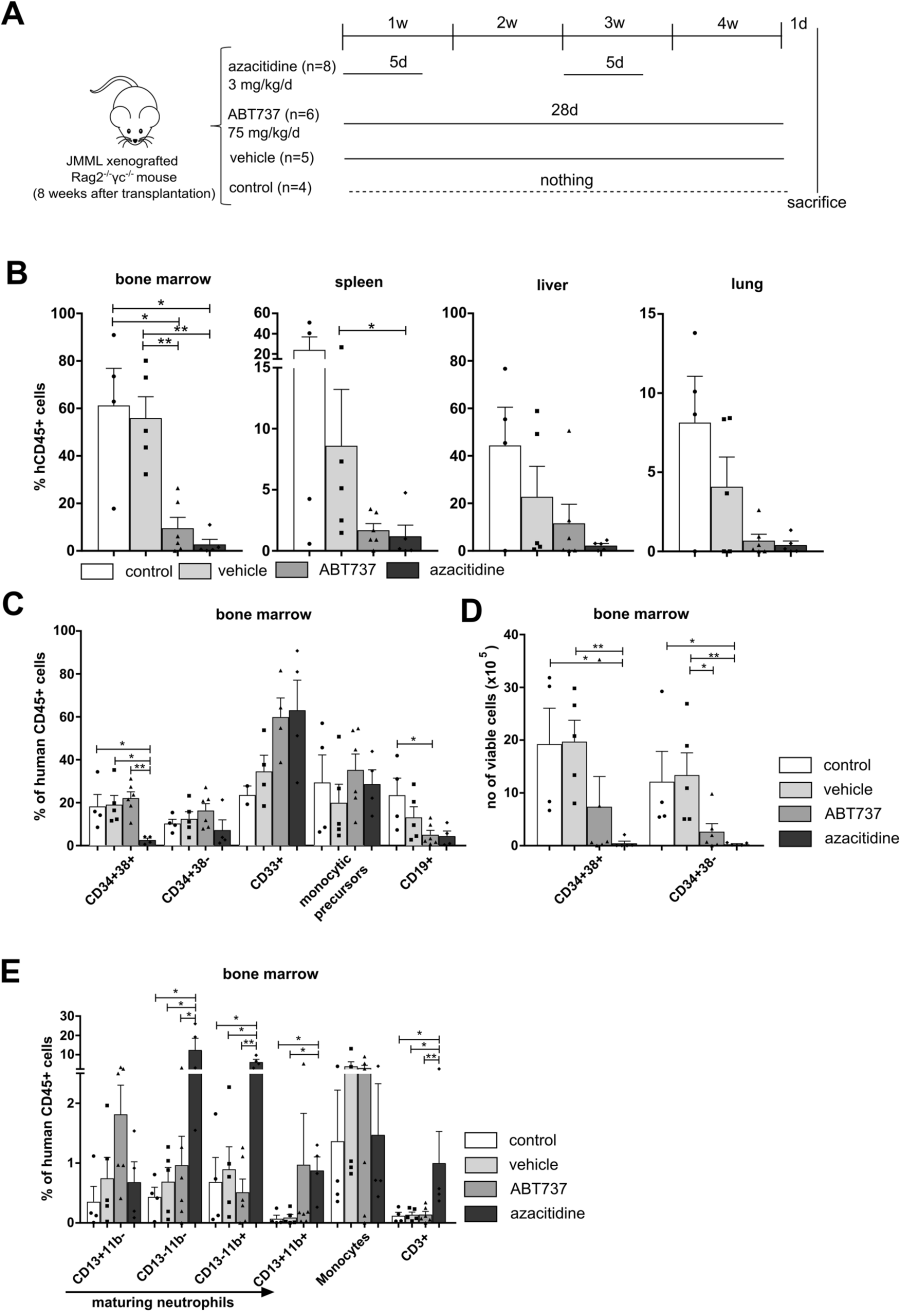
JMML cells are effectively eliminated by both ABT737 and azacitidine in a patient-derived xenograft model. **A** Spleen mononuclear cells (MNC) from JMML patients were transplanted into sublethally irradiated *Rag2*^*−/−*^*γc*^*−/−*^ mice. After 8 weeks of leukemic expansion, xenografted mice were treated with ABT737 (75 mg/kg/d, dissolved in 90% vehicle, administered daily for 4 weeks intraperitoneally). For comparison, mice were treated with azacitidine (3 mg/kg/d, dissolved in NaCl, administered intraperitoneally for two cycles of 5 consecutive days every two weeks, followed by 9 days rest afterwards). Control groups included mice treated with vehicle (dimethyl sulfoxide: vehicle, which is 25 mM HCl with 20% 2-hydroxypropyl-β-cyclodextrin = 1:9) or were left untreated (control). Twenty mice were alive after the treatment and were analyzed 4 weeks after start of therapy. **B** Hematopoietic cells were obtained from indicated organs (bone marrow (BM) from two femurs) one day after the last treatment and human cell engraftment was assessed by flow cytometry using hCD45 antibody. **C**–**E** JMML cell subpopulations were determined by flow cytometry and absolute cell counts (**D**) in each population were calculated in BM (both femurs). CD34^+^CD38^-^: immature hematopoietic stem and progenitor cells, CD34^+^CD38^+^: more mature hematopoietic progenitor cells, CD33^+^: myeloid cells, CD19^+^: B cells, CD3^+^: T cells. Bars represent mean ± standard error of mean (SEM). *n* = 4 from four independent experiments. Mann Whitney: *p < 0.05, **p < 0.01.

In order to compare the antileukemic efficacy of the BCL-2/BCL-X_L_/BCL-W inhibitor ABT737 and the hypomethylating agent azacitidine, both compounds were tested in *Rag2*^*−/−*^*γc*^*−/−*^ mice xenografted with human JMML. We have previously established this patient-derived xenograft (PDX) model which presents typical disease features and allows long-term expansion of primary human JMML cells [6]. We also demonstrated that all leukemic cells originating in xenografted mice present the leukemia-initiating PTPN11 mutation and stably retain their epigenetic signature [7]. Eight weeks after transplantation, mice were allocated to the treatment groups based on % human cells in peripheral blood in order to use comparable levels of human CD45^+^ engraftment. Treatment with ABT737 or azacitidine was performed as shown in Fig. 1A. Briefly, JMML PDX mice were treated with ABT737 75 mg/kg/d daily for four weeks, a concentration frequently used for in vivo experiments [12]. Azacitidine was administered at a concentration of 3 mg/kg/d on days 1 to 5 in two 14-day cycles, a regimen adapted from recent clinical trials [8]. Both treatment with ABT737 and azacitidine markedly reduced the leukemic burden as reflected by a reduced rate of human CD45^+^ cells in all analyzed organs, with the strongest depletion detected in bone marrow (Fig. 1B). In direct comparison, azacitidine tended to be more effective than the BH3 mimetic ABT737 in targeting JMML cells. Analysis of the human JMML subpopulations in bone marrow revealed that azacitidine mainly decreased the immature hematopoietic stem and progenitor cell (HSPC) compartment (Fig. 1C). The depletion of immature HSPCs was also demonstrated by reduced absolute cell counts of CD34^+^38^+^ and CD34^+^38^-^ cells in bone marrow (Fig. 1D). ABT737 significantly decreased the number of CD34^+^38^-^ cells in bone marrow but to a lesser extent than azacitidine (*p* values: control vs. azacitidine *:0.015; vehicle vs. ABT737 *:0.017; vehicle vs. azacitidine **:0.008). Confirming earlier observations [7], azacitidine treatment resulted in increased numbers of myeloid cells, specifically neutrophil precursors and mature neutrophils (Fig. 1E).

### Efficient and sustained reduction of leukemia burden by combined treatment with ABT737 and low-dose azacitidine

**Fig. 2:**
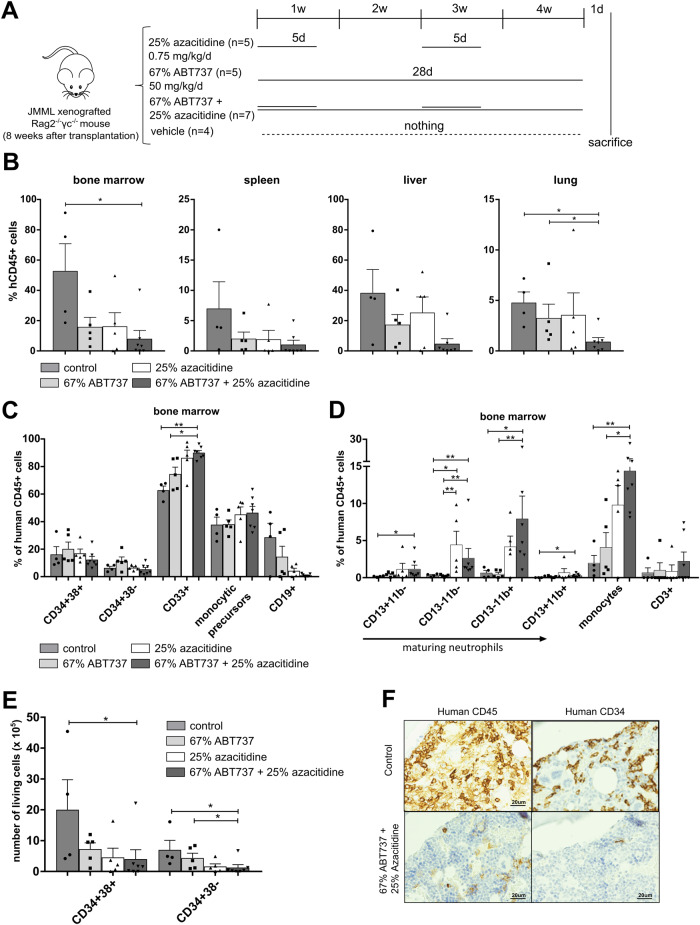
The combination of ABT737 and low-dose azacitidine efficiently reduces the leukemic burden in vivo. **A** Spleen mononuclear cells (MNC) from JMML patients were transplanted into sublethally irradiated *Rag2*^*−/−*^*γc*^*−/−*^ mice. After 8 weeks of leukemic expansion, xenografted mice were treated with either 25% azacitidine (0.75 mg/kg/d, dissolved in NaCl, administered intraperitoneally for two cycles of 5 consecutive days every two weeks, followed by 9 days rest afterwards), 67% ABT737 (50 mg/kg/d, dissolved in 90% vehicle, administered daily for 4 weeks intraperitoneally) or with a combination of both drugs. Control mice were treated with the appropriate vehicle. Mice were treated for 4 weeks and sacrificed for analysis one day after the last treatment. **B** Hematopoietic cells were obtained from indicated organs (bone marrow (BM) from two femurs) one day after the last treatment and human cell engraftment was assessed by flow cytometry using hCD45 antibody. **C**–**E** JMML cell subpopulations were determined by flow cytometry and absolute cell counts (**D**) in each population were calculated in BM (both femurs). CD34^+^CD38^−^: immature hematopoietic stem and progenitor cells, CD34^+^CD38^+^: more mature hematopoietic progenitor cells, CD33^+^: myeloid cells, CD19^+^: B cells, CD3^+^: T cells. Bars represent mean ± SEM. *n* = 4–7 from five independent experiments. Mann Whitney: * *p* < 0.05, ** *p* < 0.01. **F** Sterna were collected for histology and immunohistochemistry after treatment and stained with anti-human CD45 or CD34 antibodies. Human cells appear brown and murine cells unstained. Sternum at 40×.

As a next step, the short-term effects of the combination of ABT737 and azacitidine were evaluated in the PDX JMML model. The dose of azacitidine was reduced to 0.75 mg/kg/d (25% of the former dose) in order to allow additive effects by the combination therapy. For the same reason, the dose of ABT737 was also reduced to 50 mg/kg/d (67% of the dose used earlier). Apart from these dosage adjustments the scheme of drug administration remained unchanged (Fig. 2A). As intended, low-dose azacitidine was less effective in reducing JMML leukemia burden compared to the formerly used higher concentration. In diminished doses, both azacitidine and ABT737 decreased the human CD45^+^ fraction to a similar extent. The combination of both drugs targeted JMML cells more effectively than the monotherapy in all analyzed organs (Fig. 2B). More detailed analysis of the JMML subpopulations in bone marrow revealed an increase of the total myeloid cell population as well as of neutrophil precursors, mature neutrophils, and monocytes in mice receiving the combination of azacitidine and ABT737 indicating that lower-dose azacitidine retained its differentiating effect on JMML cells (Fig. 2C, D). Most importantly, the absolute counts of immature CD34^+^38^+^ and CD34^+^38^-^ HSPCs were significantly reduced by the combination therapy (Fig. 2E; p-values: control vs. ABT737 + azacitidine *:0.024; control vs. ABT737 + azacitidine *:0.042; ABT737 vs. ABT737 + azacitidine *:0.48). Histology and immunohistochemistry of the sternum indicated that the bone marrow was strongly infiltrated with human CD45^+^ leukemic cells and contained many immature human CD34^+^ cells. Combined treatment with ABT737 and azacitidine almost completely eliminated human cell infiltrates (Fig. 2F, Supplementary Fig. 2A–C).

**Fig. 3:**
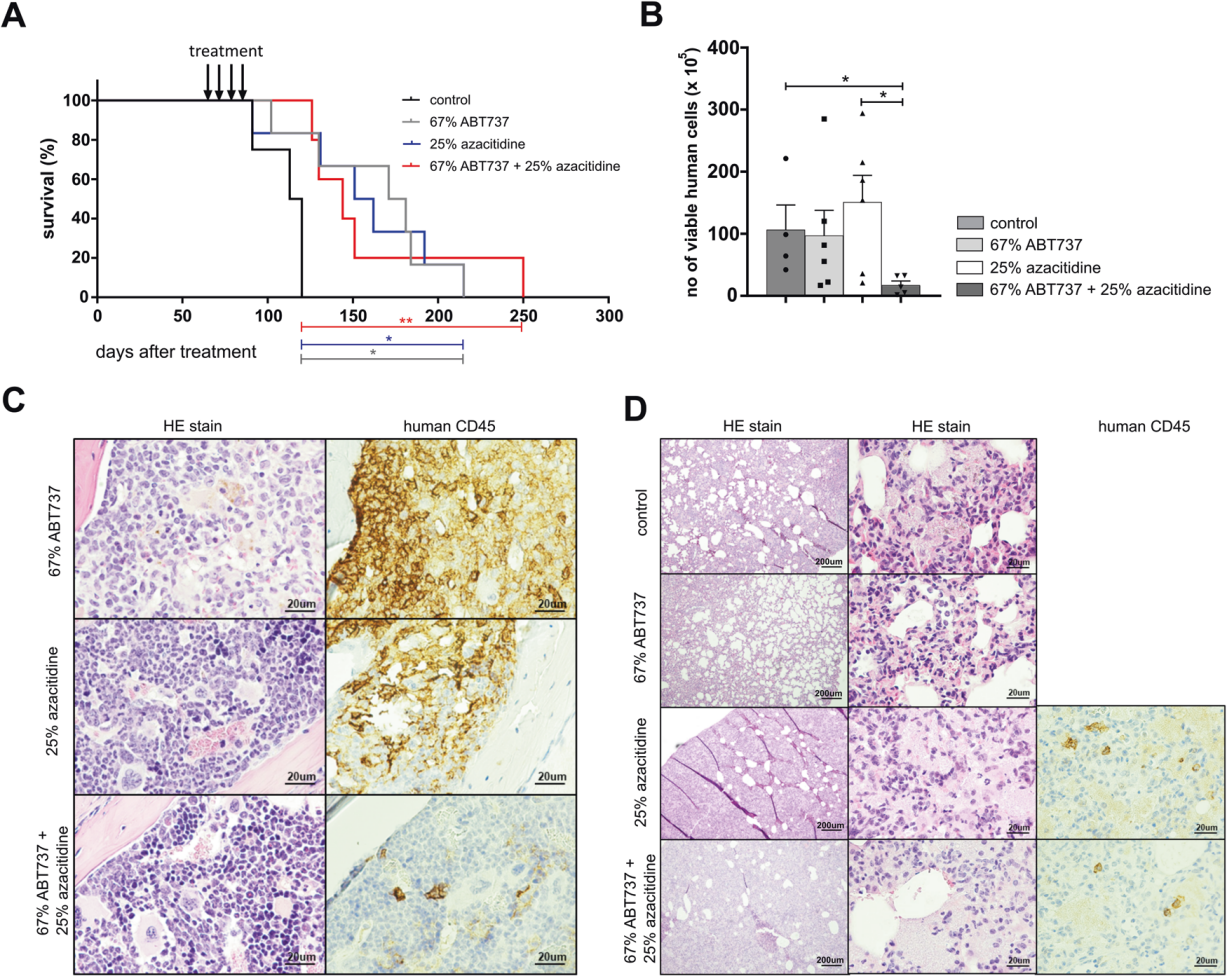
Survival of JMML xenografted mice is prolonged by both ABT737 and azacitidine. **A** Treatment regimen of the xenografted mice was identical to Fig. 2. Mice either died spontaneously or were sacrificed when their physical condition exacerbated. Kaplan-Meier-Plot showing survival rates after different treatments. Log-rank Mantel–Cox: * *p* < 0.05, ** *p* < 0.01. When terminal, hematopoietic cells were obtained from indicated organs (bone marrow from two femurs). **B** Human cell engraftment was assessed by flow cytometry using hCD45 antibody and absolute cell count was calculated. *n* = 4–6 from five independent experiments. Bars represent mean ± SEM. Mann Whitney test * *p* < 0.05, ** *p* < 0.01. Sterna and lungs were collected for histology and immunohistochemistry when terminal in each group and stained with HE and anti-human CD45 antibody. Human cells appear brown and murine cells unstained. **C** Sternum at 40×. **D** Lung at 4× and 40×.

To determine the long-term effects of the combination treatment with ABT737 and azacitidine, the survival of mice assigned to the different treatment groups was analyzed. Mice were treated for four weeks as shown in Fig. 2A and then closely monitored for signs of leukemia. All treatments significantly prolonged the survival of JMML xenografted mice (*p* values: control vs. ABT737 *:0.023; control vs. azacitidine *:0.024; control vs. ABT737 + azacitidine **<:0.01). However, differences between combined treatment and either drug in monotherapy were not observed (Fig. 3A). Eventually, all animals died between 130 and 200 days after xenotransplantation. Analysis of the fraction of human and murine cells in bone marrow at terminal disease stage revealed that all animals that had received no therapy or monotherapy died from leukemia. In contrast, the 4-week treatment with the combination of ABT737 and azacitidine resulted in a sustained depletion of human JMML cells (Fig. 3B). In line with these results, histology and immunohistochemistry of the sternum of terminal animals showed that only the combined treatment reduced the human JMML infiltrates in bone marrow (Fig. 3C, Supplementary Fig. 3B–E). Few human CD45^+^ cell infiltrates were detected in the lungs of animals that had received the combination therapy (Fig. 3D). Yet, the pulmonary tissue structure appeared severely damaged in animals treated with azacitidine, either as mono- or combination therapy. This might explain why animals receiving the combination treatment died despite successful elimination of leukemic infiltrates.

### Leukemia-initiating cells are depleted by combined treatment with ABT737 and low-dose azacitidine

**Fig. 4:**
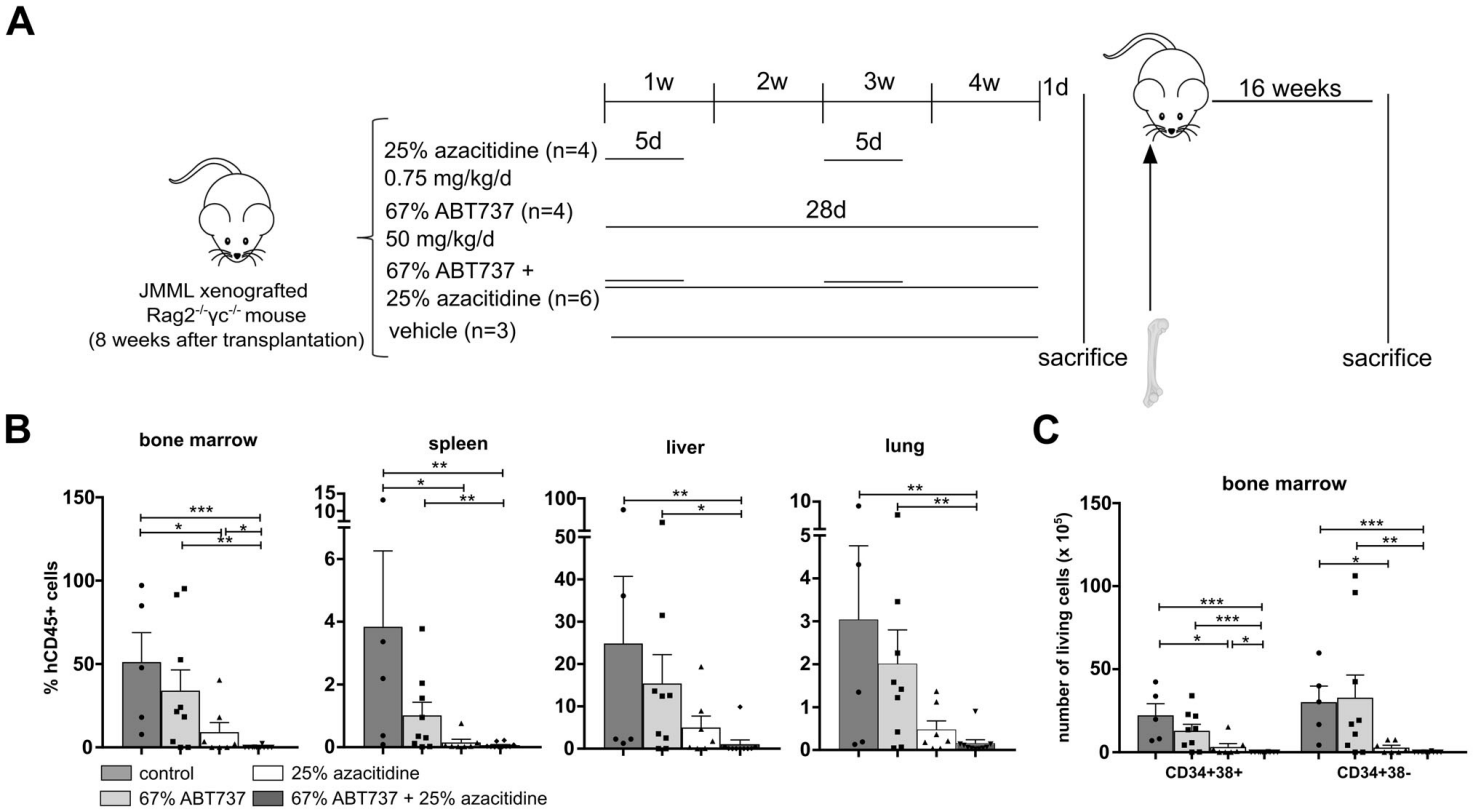
ABT737 and low-dose azacitidine are able to eliminate leukemia-initiating JMML cells. **A** Spleen mononuclear cells (MNC) from JMML patients were transplanted into sublethally irradiated *Rag2*^*−/−*^*γc*^*−/−*^ mice. After 8 weeks of leukemic expansion, xenografted mice were treated with either 25% azacitidine (0.75 mg/kg/d, dissolved in NaCl, administered intraperitoneally for two cycles of 5 consecutive days every two weeks, followed by 9 days of rest afterwards), 67% ABT737 (50 mg/kg/d, dissolved in 90% vehicle, administered daily for 4 weeks intraperitoneally) or with a combination of both drugs. Control mice were treated with the appropriate vehicle. Mice were treated for 4 weeks and sacrificed one day after the last treatment. Bone marrow was isolated from those mice and transplanted into syngeneic secondary recipient mice. **B** Secondary transplanted mice were sacrificed 16 weeks after serial transplantation and hematopoietic cells were obtained from indicated organs (bone marrow (BM) from two femurs, spleen, liver, and lung). Human engraftment was assessed by flow cytometry using hCD45 antibody. **C** JMML cell subpopulations were determined by flow cytometry and absolute cell counts in each population were calculated in BM (both femurs). CD34^+^CD38^−^: immature hematopoietic stem and progenitor cells, CD34^+^CD38^+^: more mature hematopoietic progenitor cells. Bars represent mean ± SEM. *n* = 5–10 from three independent experiments. Mann Whitney test: * *p* < 0.05, ** *p* < 0.01, *** *p* < 0.001.

As shown above, stem and progenitor cells were efficiently depleted by the combination of ABT737 and low-dose azacitidine. In order to assess this effect also on a functional level, we performed serial transplantations (Fig. 4A) based on the hypothesis that JMML cells from animals that had received combined treatment would not be able to induce leukemia in secondary recipient mice. Engraftment of JMML cells was analyzed 16 weeks after the second transplantation (Fig. 4B). In direct comparison, azacitidine monotherapy reduced JMML engraftment in serially transplanted recipient animals more effectively than ABT737. However, all recipients serially transplanted with JMML cells treated with either ABT737 or azacitidine showed JMML engraftment indicating that sufficient numbers of leukemia initiating cells survived the monotherapy. In contrast, the combination of both treatments was more potent and resulted in sufficient depletion of JMML-initiating cells: nine of ten secondary recipients did not show JMML engraftment (Fig. 4C).

### Non-selective BCL-2/BCL-X_L_/BCL-W and selective BCL-X_L_ inhibitors exert the best synergism with azacitidine against JMML in vitro

**Fig. 5:**
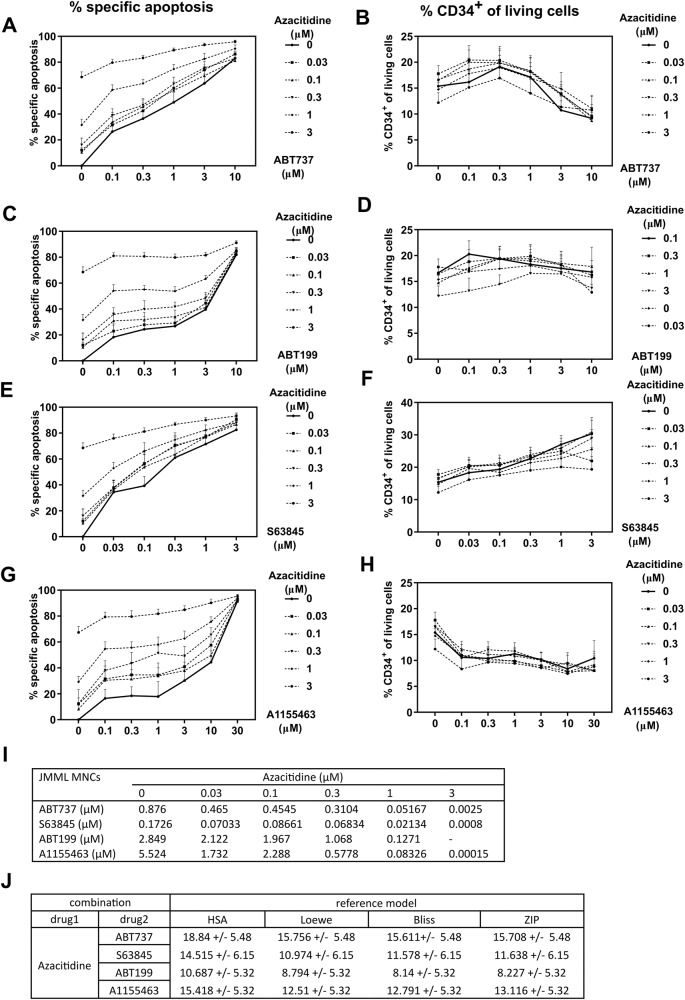
The BH3 mimetics ABT737 and A1155463 synergize best with azacitidine to target JMML cells in vitro. MNCs from JMML patients were treated with increasing doses of azacitidine (0.03, 0.1, 0.3, 1, 3 µM) in combination with either ABT737 (0.1, 0.3, 1, 3, 10 µM), ABT199 (0.1, 0.3, 1, 3, 10 µM), S63845 (0.03, 0.1, 0.3, 1, 3 µM) or A1155463 (0.1, 0.3, 1, 3, 10, 30 µM) to determine synergism between the drugs. **A**, **C**, **E**, **G** Apoptosis was measured 72 h after treatment using Annexin V/7-AAD. **B**, **D**, **F**, **H** The proportion of CD34^+^ cells was analyzed by flow cytometry (percentage of human CD34^+^ among all living human cells). Graphs represent mean ± SEM. *n* = 6–7 from seven independent experiments. **I** IC50 and **J** dose response matrix of inhibition were determined using the web application SynergyFinder (https://synergyfinder.fimm.fi/) on JMML MNCs after the treatment of each BH3 mimetic and azacitidine.

We had previously decided to use the BH3 mimetic ABT737 due to its broad spectrum targeting BCL-2, BCL-X_L_, and BCL-W. As a next step, we tested selective BCL-2 protein inhibitors in combination with azacitidine in order to identify the individual contribution of each antiapoptotic BCL-2 protein to the survival of JMML cells. Killing efficacy in primary JMML cells and drug synergy with azacitidine was tested for the BH3 mimetics ABT737 (inhibitor of BCL-2, BCL-X_L_, and BCL-W), ABT199 (selective BCL-2 inhibitor), S63845 (MCL-1 inhibitor), and A1155463 (BCL-X_L_ inhibitor). BCL-W was not inhibited selectively given that its expression in the hematopoietic system is low and *BCL-W*-deficient mice show no overt hematopoietic phenotype [20, 21]. Primary JMML mononuclear cells were cultured in vitro under previously optimized conditions [22]. Both ABT737 and S63845 induced dose-dependent apoptosis even at low concentrations, while ABT199 and A1155463 were similarly effective only at higher doses (Fig. 5A, C, E, and G). All BH3 mimetic drugs induced apoptosis to a greater extent when used in combination with azacitidine. As monotherapy, the MCL-1 inhibitor S63845 had the lowest IC50 value followed by ABT737 and ABT199, while the BCL-X_L_ inhibitor A1155463 showed the highest IC50 value (Fig. 5I). All IC50 values decreased when combining the BH3 mimetics with escalating doses of azacitidine. Of note, azacitidine alone was also effective to induce apoptosis in primary JMML cells. To quantify the synergy of the different combinations of BH3 mimetics with azacitidine, summary synergy scores were calculated according to several reference models (Fig. 5J). Synergism, defined by summary synergy scores over 10, was observed for the combination of azacitidine with ABT737, S63845, and A1155463. The summary synergy scores ranging from −10 to 10 indicated additive effects of azacitidine and ABT199. The highest scores and thus the best synergistic effects were detected for the combination of azacitidine with A1155463, directly followed by the combination with ABT737, indicating that BCL-X_L_ is an essential survival protein for JMML cells under azacitidine treatment. We also investigated how these drug combinations influenced the amount of leukemia-initiating cells in this in vitro culture system (Fig. 5B, D, F, and H). Monotherapy with both ABT737 and A1155463 diminished the proportion of human CD34^+^ cells in a dose-dependent manner. Treatment with ABT199 had no effect on the percentage of immature JMML cells, while the MCL-1 inhibitor S63845 even caused a slight increase in this cell population. Additional treatment with azacitidine only mildly influenced the proportion of human CD34^+^ cells. In summary, both the non-selective BCL-2/BCL-X_L_ inhibitor ABT737 and the selective BCL-X_L_ inhibitor A1155463 exerted synergism to kill JMML cells in combination with azacitidine, and additionally targeted leukemia-initiating cells better than azacitidine.

### MCL-1 and BCL-X_L_ are essential for the survival of JMML cells

**Fig. 6:**
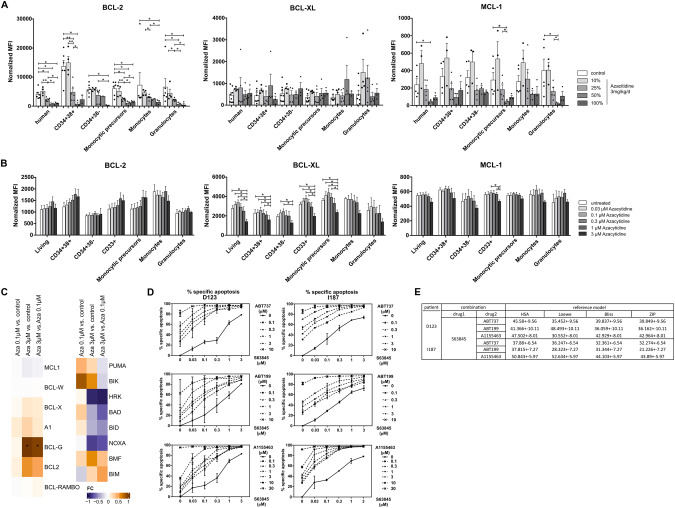
JMML cells critically depend on MCL-1 and BCL-X_L_ for survival. **A** Spleen mononuclear cells (MNC) from JMML patients were transplanted into sublethally irradiated *Rag2*^*-/-*^*γc*^*-/-*^ mice. After 8 weeks of leukemic expansion, xenografted mice were treated with different doses of azacitidine or were left untreated as control. One day after the last injection murine bone marrow (BM) was collected and the intracellular expression of the BCL-2 family members BCL-2, MCL-1 and BCL-X_L_ was determined by flow cytometry in each subpopulation. The expression was determined as the normalized mean fluorescence intensity (MFI). CD34^+^CD38^-^: immature hematopoietic stem and progenitor cells, CD34^+^CD38^+^: more mature hematopoietic progenitor cells. Bars represent mean ± SEM. *n* = 3–7 from four independent experiments. Mann Whitney: * *p* < 0.05, ** *p* < 0.01. **B** JMML MNCs were treated with azacitidine (0.03, 0.1, 0.3, 1 and 3 μM) for 72 h and the intracellular expression of BCL-2, BCL-X_L_ and MCL-1 was determined by flow cytometry in each subpopulation. The expression was determined as the normalized mean fluorescence intensity (MFI). Bars represent mean ± SEM. *n* = 4 from four independent experiments. Mann Whitney: * *p* < 0.05. **C** Human cells were purified from xenotransplanted JMML mouse BM after 12 weeks of leukemic expansion, subsequently treated with azacitidine (0, 0.1, or 3 µM) for 72 h ex vivo and then collected for RNA sequencing. Heatmap illustrates the pairwise comparisons between azacitidine (0.1 or 3 µM) and control, as labeled on top. Color code represents the log2 fold-change (fc). **D** MNCs from two different JMML patients (D123, I187) were treated with increasing doses of the inhibitor S63845 in combination with either ABT737, ABT199, or A1155463 to determine synergism between the drugs. Apoptosis was measured 24 h after treatment using Annexin V/7-AAD. The percentage of specific apoptosis was calculated. **E** A dose–response matrix and summary synergy scores of each BH3 mimetic and azacitidine were determined using the web application SynergyFinder (https://synergyfinder.fimm.fi/). (*n* = 2 from two independent experiments per patient).

We next aimed to further clarify the interactions between BCL-2 protein inhibitors and azacitidine in JMML. We hypothesized that azacitidine increased or reduced the levels of pro- or antiapoptotic BCL-2 proteins, respectively. As a first step, the protein levels of the antiapoptotic BCL-2 family members BCL-2, BCL-XL, and MCL-1 were determined by intracellular staining in JMML mononuclear cells after azacitidine treatment of xenografted mice. In all analyzed subpopulations, the BCL-2 protein levels were significantly decreased by azacitidine in a dose-dependent way (Fig. 6A). A similar effect was observed for MCL-1. BCL-XL levels were not affected by azacitidine treatment in vivo. In complementary in vitro experiments, we analyzed the amount of these prosurvival BCL-2 proteins in JMML cells incubated with azacitidine at different concentrations ex vivo (Fig. 6B). In this setting, BCL-2 levels were lower than in vivo and not influenced by azacitidine treatment. In contrast, BCL-XL levels were significantly reduced by azacitidine. MCL-1 protein levels were only mildly diminished. We also studied the expression of proapoptotic BH3-only proteins on the RNA level after in vitro treatment with azacitidine. Low doses of azacitidine resulted in a high upregulation of BIK and a mild upregulation of PUMA, BAD and BMF mRNA levels. Higher doses of azacitidine either induced upregulation of BIM and further increased upregulation of BMF or resulted in downregulation of all the other BH3-only proteins on the transcriptional level. Prosurvival proteins BCL-2 and BCL-G were upregulated on mRNA level in a dose-dependent manner (Fig. 6C). Expression of the proapoptotic proteins BAX and BAK were mildly downregulated (Supplementary Fig. 4). In sum, these data indicate that azacitidine treatment shifts the balance towards a proapoptotic state both in vitro and in vivo. While proapoptotic BH3-only proteins are upregulated on mRNA level, the antiapoptotic proteins are downregulated by post-translational mechanisms.

In order to elucidate the antiapoptotic role of the different prosurvival BCL-2 proteins irrespective of azacitidine treatment, we analyzed possible synergistic effects of different BH3 mimetics by combining ABT737 (inhibitor of BCL-2, BCL-XL, and BCL-W), ABT199 (selective BCL-2 inhibitor), S63845 (MCL-1 inhibitor), and A1155463 (BCL-XL inhibitor) in vitro. All combinations were synergistic and shifted the equilibrium towards cell death, but especially combined treatment with S63845 and ABT737 or A-1155463 showed strong synergism in both patient samples, indicating that concomitant inhibition of both MCL-1 and BCL-XL is synergistically lethal in JMML (Fig. 6D, E).

### No synergy was observed in the combination of low-dose azacitidine and ABT199

**Fig. 7:**
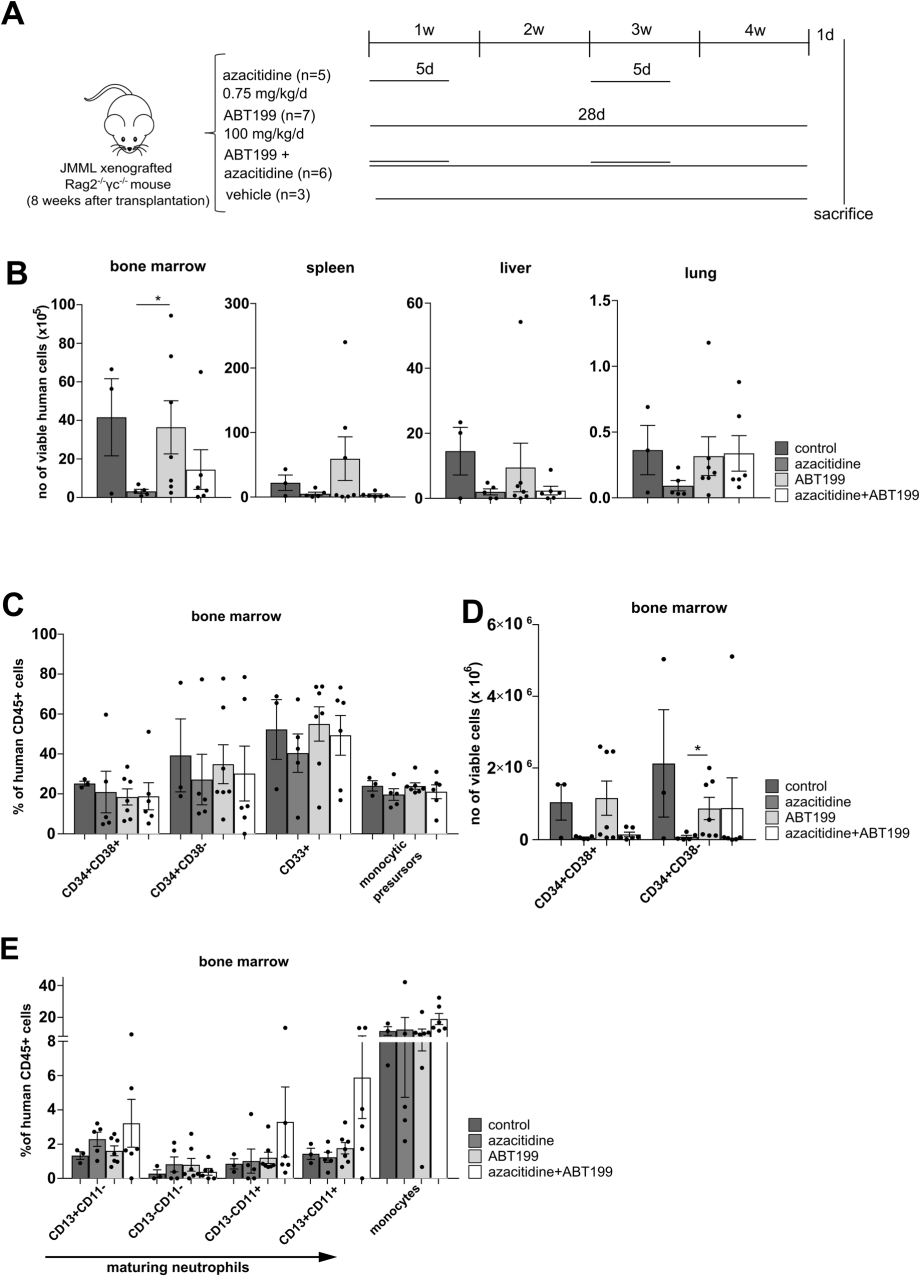
ABT199 is inferior to ABT737 in eliminating leukemia-initiating JMML cells. **A** Spleen mononuclear cells (MNC) from JMML patients were transplanted into sublethally irradiated *Rag2*^*−/−*^*γc*^*−/−*^ mice. After 8 weeks of leukemic expansion, xenografted mice were treated with either azacitidine (0.75 mg/kg/d, dissolved in NaCl, administered intraperitoneally for two cycles of 5 consecutive days every two weeks, followed by 9 days of rest afterwards), ABT199 (100 mg/kg/d, dissolved in vehicle, administered daily for 4 weeks by oral gavage) or with a combination of both drugs. Control mice were treated with the appropriate vehicle. Mice were treated for 4 weeks and sacrificed one day after the last treatment. **B** Hematopoietic cells were obtained from indicated organs (bone marrow (BM) from two femurs) one day after the last treatment and human cell engraftment was assessed by flow cytometry using hCD45 antibody. Organs were counted and absolute cell counts were calculated. **C**–**E** JMML cell subpopulations were determined by flow cytometry and absolute cell counts (**D**) in each population were calculated in BM (both femurs). CD34^+^CD38^-^: immature hematopoietic stem and progenitor cells, CD34^+^CD38^+^: more mature hematopoietic progenitor cells, CD33^+^: myeloid cells. Bars represent mean ± SEM. *n* = 3–7 from three independent experiments. Mann Whitney: * *p* < 0.05.

The specific BCL-2 inhibitor venetoclax (ABT199) is the only FDA-approved BH3 mimetic so far, and its combination with azacitidine works efficiently in other myeloid neoplasias [23]. Despite the limited synergistic action of venetoclax and azacitidine in vitro, we tested this combination in our PDX model. Mice were treated according to the scheme depicted in Fig. 7A with low-dose azacitidine, ABT199 100 mg/kg/d administered daily by oral gavage or the combinatorial treatment. The effects of ABT199, however, were underwhelming. ABT199 monotherapy had no influence on the leukemic burden in all analyzed organs nor on the JMML subpopulations in bone marrow (Fig. 7B-E). Also in combination with low-dose azacitidine no clear synergistic effects could be observed.

## Discussion

JMML is a rare pediatric neoplasia, which requires allogeneic HSCT for a curative treatment option in many cases. Still, transplantation-related morbidity and a relevant risk of disease recurrence after HSCT highlight the need for innovative therapeutic approaches [24]. Most JMML cases are characterized by a constitutively active RAS/MAPK signaling pathway, which in turn promotes cell survival via interference with the physiological apoptosis pathways [11, 3]. Effects of a constitutively active RAS/MAPK signaling pathway are cell type-specific and include activation of BCL-2, MCL-1, and BCL-X_L_ as well as inactivation of BAD, BIM, and NOXA [25–28]. While apoptosis resistance certainly is a hallmark of cancer that contributes to neoplastic transformation [29], it is also known that cytotoxic drugs induce apoptosis more easily in cancer cells than in non-malignant tissues. Despite their advantage in vivo, many cancer cells appear to proceed to a status of “mitochondrial priming” rendering them susceptible to treatment with BH3 mimetics [30–32]. Based on these considerations, we decided to evaluate the BH3 mimetic ABT737, which is a broad-spectrum BCL-2 protein inhibitor, in comparison with azacitidine as best available therapy in our preclinical JMML model. Both treatments resulted in the depletion of JMML cells, but azacitidine targeted immature HSPCs more selectively and to a greater extent than ABT737.

The efficacy of the DNA methyltransferase inhibitor azacitidine as pre-transplant therapy has already been proven in several clinical trials with JMML patients but drug resistance still remains a major problem [33]. Different mechanisms contribute to resistance to hypomethylating agents including deregulation of apoptotic pathways by changes of BCL-2 protein levels [34]. The combination of low-dose azacitidine and ABT737 reduced the leukemic burden more effectively in the PDX JMML model than either compound alone, and retained the differentiating effect of azacitidine on JMML cells. The overall survival of animals receiving the combination, however, did not differ from the other treatment groups. Histologic analyses revealed severe damages of the pulmonary tissue structure, presumably reflecting pulmonary edema, in the mice that had received azacitidine. This effect in the murine system has not been described so far but we assume that it was the leading cause of death. Robust clinical data demonstrate the safety of azacitidine therapy in humans, with myelosuppression as the most common adverse event [35]. We therefore conclude that the observed side effect is apparently mouse-specific and has no relevance for an application in humans. Navitoclax, the orally bioavailable derivative of ABT737, had not obtained FDA approval due to dose-limiting thrombocytopenia noticed in phase I studies, an effect that could be attributed to BCL-X_L_ inhibition [36–38]. In our PDX JMML model, we did not observe altered platelet levels but still achieved good antitumor effectivity. Combined treatment with azacitidine and ABT737 enabled us to lower the doses of both drugs and hence reduce undesired side effects while still being able to successfully deplete leukemia-initiating cells. Various cycles of this combination therapy might be required to improve clinical response rates and control JMML in the long term.

Our in vitro experiments give insight into the individual roles of BCL-2, BCL-X_L_, and MCL-1 in primary JMML cells. Combination therapy with specific BH3 mimetics revealed that BCL-X_L_ and MCL-1 are both essential for JMML cell survival. Their combined inhibition results in synthetic lethality. Given that MCL-1 is downregulated by azacitidine in vivo, the synergistic effect of the BCL-X_L_ inhibitor and azacitidine can likely be ascribed to the combined inhibition of both proteins. While combined use of BCL-X_L_ and MCL-1 might result in excessive toxicity [39], the combination of azacitidine and BCL-X_L_ inhibition might be tolerated better. Earlier experiments in acute myeloid leukemia (AML) cell lines support our results, reporting that MCL-1 levels were downregulated by azacitidine and that this resistance mechanism could be overcome by additional BH3 mimetic treatment with ABT737 [40]. Also, the induction of the BH3-only protein NOXA by azacitidine and the consecutive priming for venetoclax-induced cell death was previously described in AML cells [41]. Accordingly, we observed azacitidine-induced upregulation of proapoptotic BH3-only proteins, which might result in increased mitochondrial priming and be the main factor contributing to the sensitivity to BH3 mimetics. It should be kept in mind, however, that the effects of azacitidine on JMML cells were different in vitro and in vivo. One possible explanation is that microenvironmental signals interfere with BCL-2 proteins in vivo. This was described earlier for CLL cells, which display increased BCL-X_L_ and MCL-1 protein levels after stromal co-culture [42]. We conclude that in vivo models such as our PDX model are crucial to determine the exact effects of drugs in JMML.

In conclusion, our findings support developing the combination of BH3 mimetics with drugs targeting the antiapoptotic proteins MCL-1 and BCL-X_L_. The combination of azacitidine with a specific BCL-X_L_ inhibitor seems to be particularly attractive since azacitidine does not only interfere with BCL-2 family homeostasis but also induces the differentiation of JMML cells to mature granulocytes.

## Conclusion and outlook

We show that JMML cells critically depend on both MCL-1 and BCL-X_L_, and that these prosurvival proteins can be successfully targeted by combined azacitidine and BH3 mimetic treatment. Azacitidine acts via downregulation of antiapoptotic MCL-1 and increased activation of proapoptotic proteins. Furthermore, our data demonstrate that BCL-X_L_ inhibition is superior to BCL-2 inhibition in eliminating JMML cells. Several BH3 mimetic drugs have been developed but the specific BCL-2 inhibitor venetoclax is the only compound with regulatory approval so far. A retrospective study has shown promising results combining azacitidine with venetoclax in children with AML or MDS [43]. Currently, more than 100 clinical studies evaluate the combination of venetoclax and azacitidine in hematological malignancies. In JMML patients, however, the effects of this combined treatment have not yet been systematically assessed so far, and our present results are not very promising. Based on the encouraging effects of ABT737, we rather support the off-label use of its analog navitoclax, which should be safely applicable given its well-known toxicity profile. While MCL-1 inhibitors are being evaluated in various hematological malignancies, selective BCL-X_L_ inhibitors have not yet entered clinical trials. Our results support the development of these compounds in combination with azacitidine in the clinical setting as soon as they are available. Still, it has to be noted that combined suppression of MCL-1 and BCL-X_L_ is already synthetically lethal in a healthy hematopoietic system so that severe cytopenias are to be expected [44, 39]. However, these adverse effects would be less significant if combined MCL-1 and BCL-X_L_ inhibition was applied as pre-transplant therapy followed by HSCT.

### Supplementary information

Supplemental data

## Data Availability

RNAseq data are deposited at GEO (GSE 242432) and available from the corresponding author on reasonable request (https://www.ncbi.nlm.nih.gov/geo/query/acc.cgi?acc=GSE242432). All other data generated or analyzed during this study are included in this published article [and its supplementary information files].
