# Supplementary material for: BH3 mimetics and azacitidine show synergistic effects on juvenile myelomonocytic leukemia
Source: Leukemia. 2023 Nov 9;38(1):136–48. doi: 10.1038/s41375-023-02079-5 (PMC10776398; doi:10.1038/s41375-023-02079-5)
Supplement: Supplementary file 1 — Supplemental data [file 41375_2023_2079_MOESM1_ESM.pptx]

## Slide 1
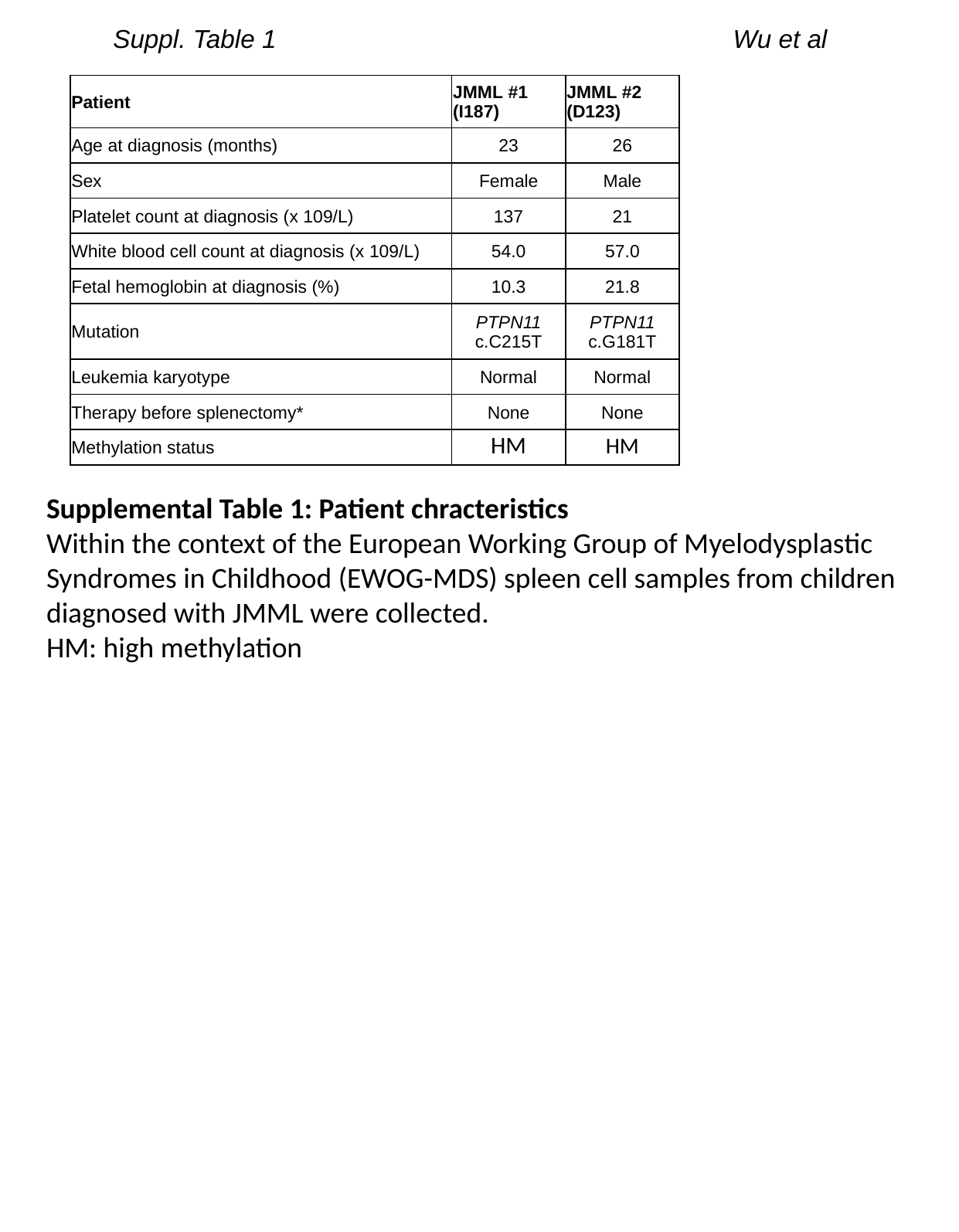

Suppl. Table 1
Wu et al
| Patient | JMML #1  (I187) | JMML #2  (D123) |
| --- | --- | --- |
| Age at diagnosis (months) | 23 | 26 |
| Sex | Female | Male |
| Platelet count at diagnosis (x 109/L) | 137 | 21 |
| White blood cell count at diagnosis (x 109/L) | 54.0 | 57.0 |
| Fetal hemoglobin at diagnosis (%) | 10.3 | 21.8 |
| Mutation | PTPN11 c.C215T | PTPN11 c.G181T |
| Leukemia karyotype | Normal | Normal |
| Therapy before splenectomy\* | None | None |
| Methylation status | HM | HM |
Supplemental Table 1: Patient chracteristics
Within the context of the European Working Group of Myelodysplastic Syndromes in Childhood (EWOG-MDS) spleen cell samples from children diagnosed with JMML were collected.
HM: high methylation

## Slide 2
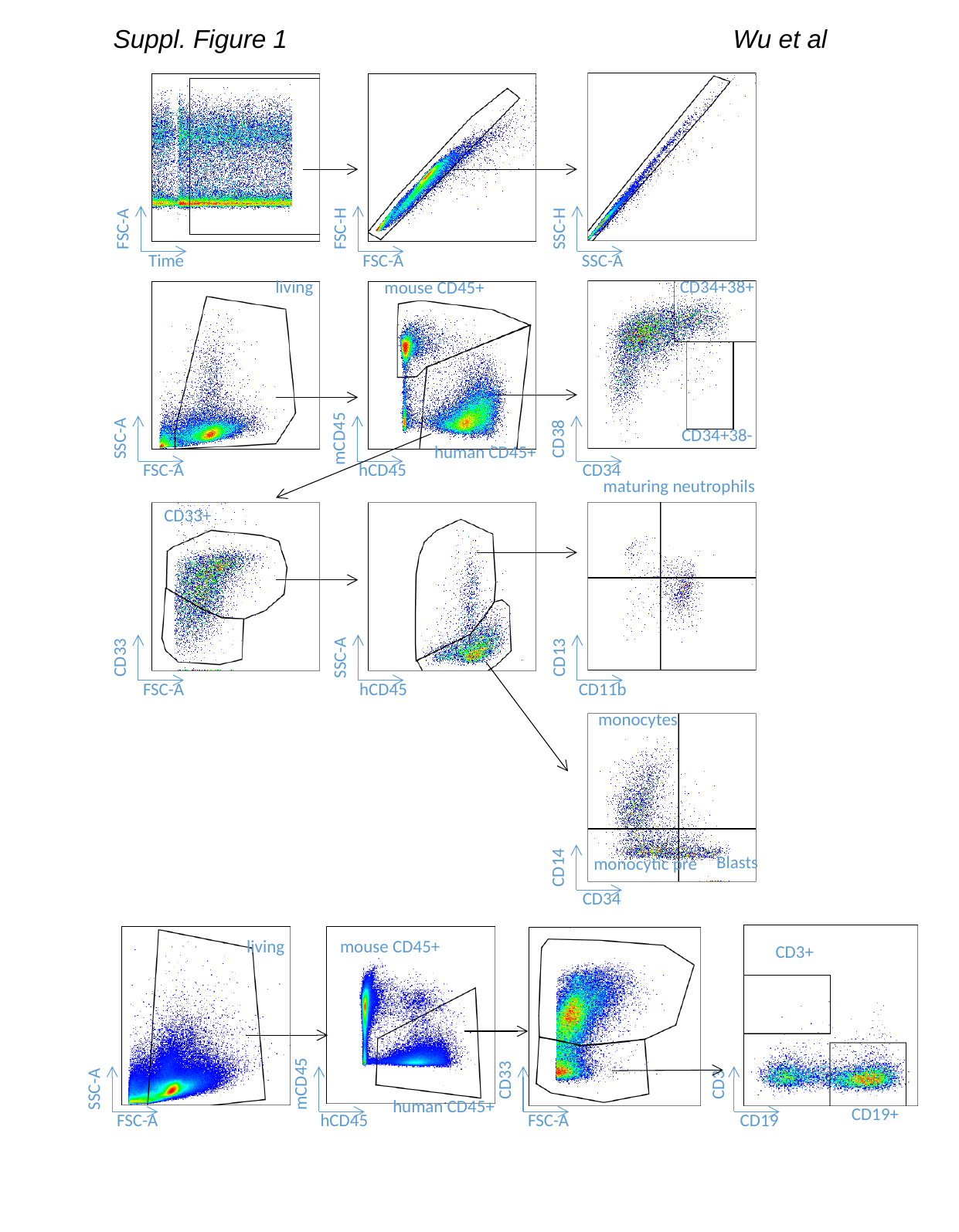

Suppl. Figure 1
Wu et al
FSC-H
SSC-H
FSC-A
mouse CD45+
FSC-A
SSC-A
Time
CD34+38+
living
CD34+38-
human CD45+
maturing neutrophils
mCD45
SSC-A
CD38
hCD45
FSC-A
CD34
CD33+
SSC-A
CD33
CD13
hCD45
CD11b
FSC-A
monocytes
monocytic pre
Blasts
CD14
CD34
mouse CD45+
living
CD3+
human CD45+
mCD45
CD33
SSC-A
CD3
CD19+
hCD45
FSC-A
FSC-A
CD19

## Slide 3
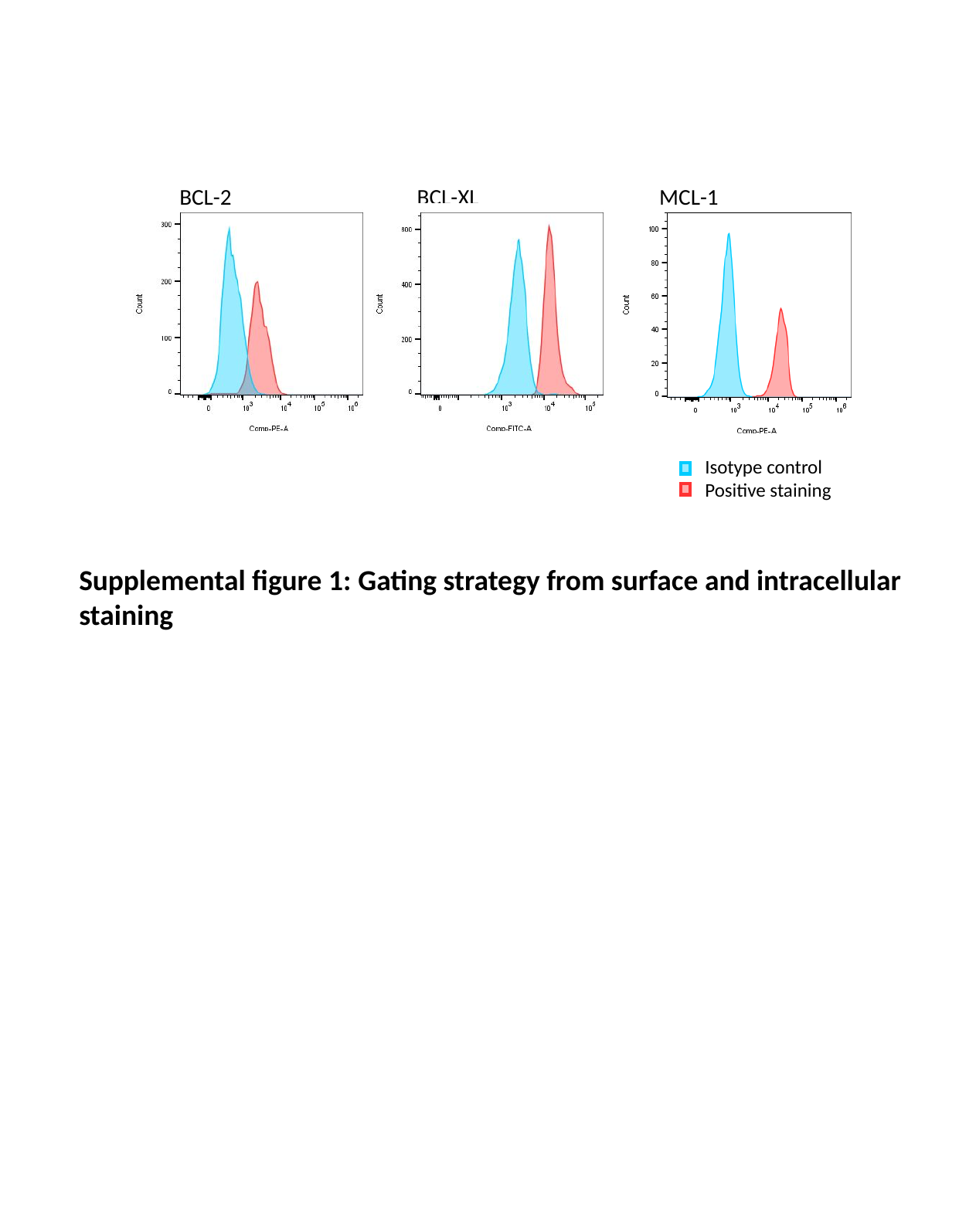

BCL-2 BCL-XL MCL-1
Isotype control
Positive staining
Supplemental figure 1: Gating strategy from surface and intracellular staining

## Slide 4
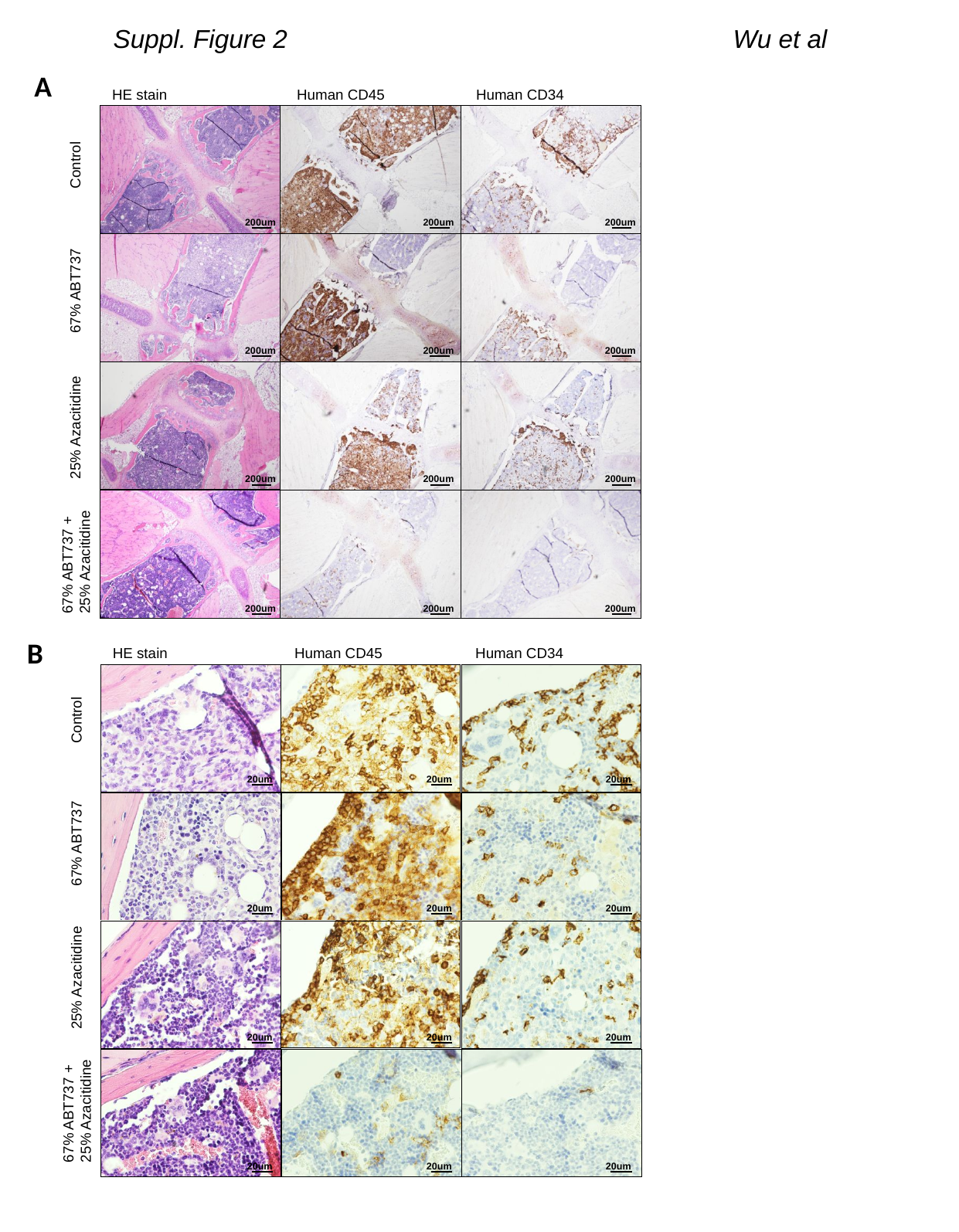

Suppl. Figure 2
Wu et al
A
HE stain
Human CD45
Human CD34
Control
67% ABT737
25% Azacitidine
67% ABT737 +
25% Azacitidine
200um
200um
200um
200um
200um
200um
200um
200um
200um
200um
200um
200um
B
HE stain
Human CD45
Human CD34
Control
20um
20um
20um
67% ABT737
20um
20um
20um
25% Azacitidine
20um
20um
20um
67% ABT737 +
25% Azacitidine
20um
20um
20um

## Slide 5
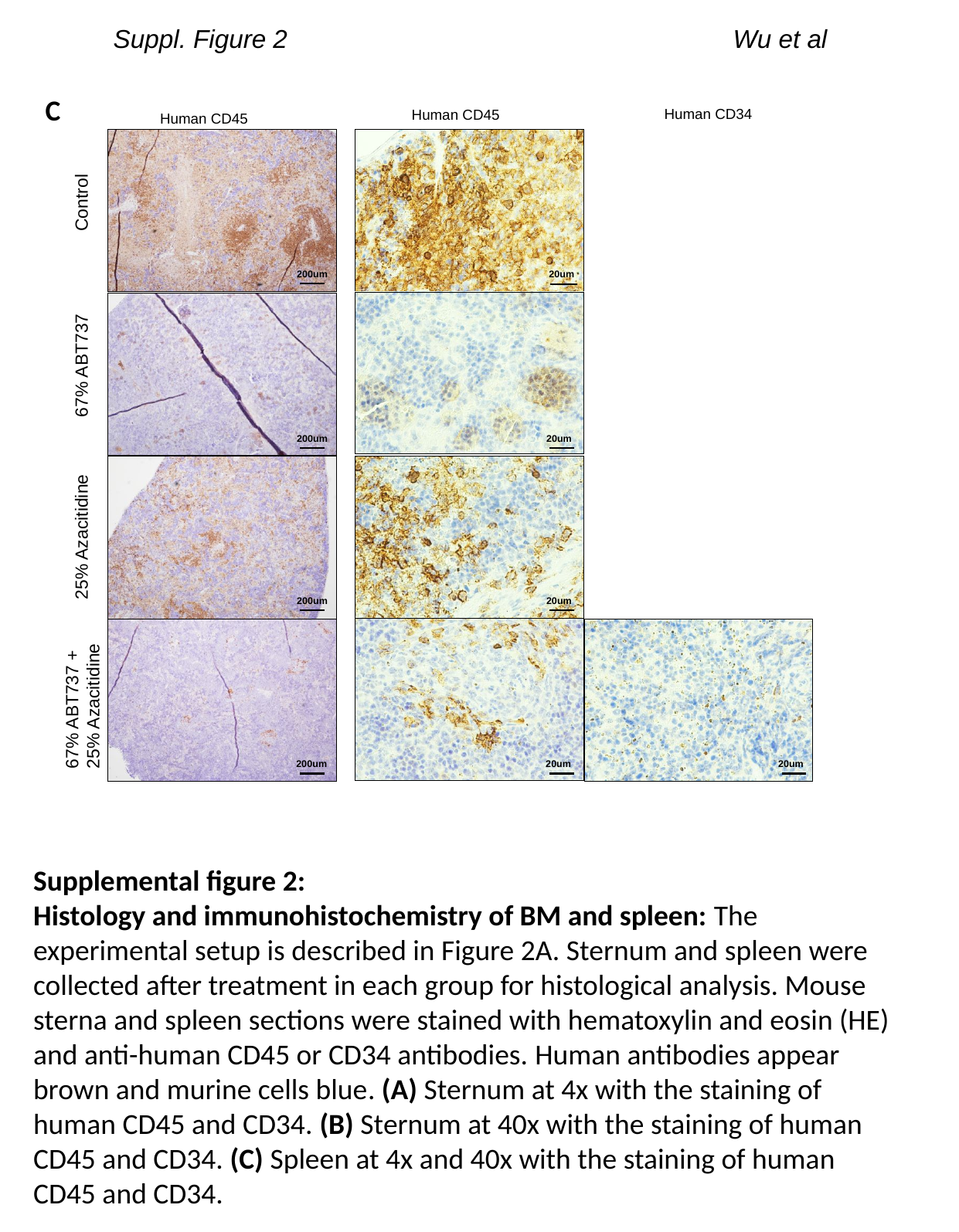

Suppl. Figure 2
Wu et al
C
Human CD34
Human CD45
Human CD45
Control
200um
20um
67% ABT737
200um
20um
25% Azacitidine
200um
20um
67% ABT737 +
25% Azacitidine
200um
20um
20um
Supplemental figure 2:
Histology and immunohistochemistry of BM and spleen: The experimental setup is described in Figure 2A. Sternum and spleen were collected after treatment in each group for histological analysis. Mouse sterna and spleen sections were stained with hematoxylin and eosin (HE) and anti-human CD45 or CD34 antibodies. Human antibodies appear brown and murine cells blue. (A) Sternum at 4x with the staining of human CD45 and CD34. (B) Sternum at 40x with the staining of human CD45 and CD34. (C) Spleen at 4x and 40x with the staining of human CD45 and CD34.

## Slide 6
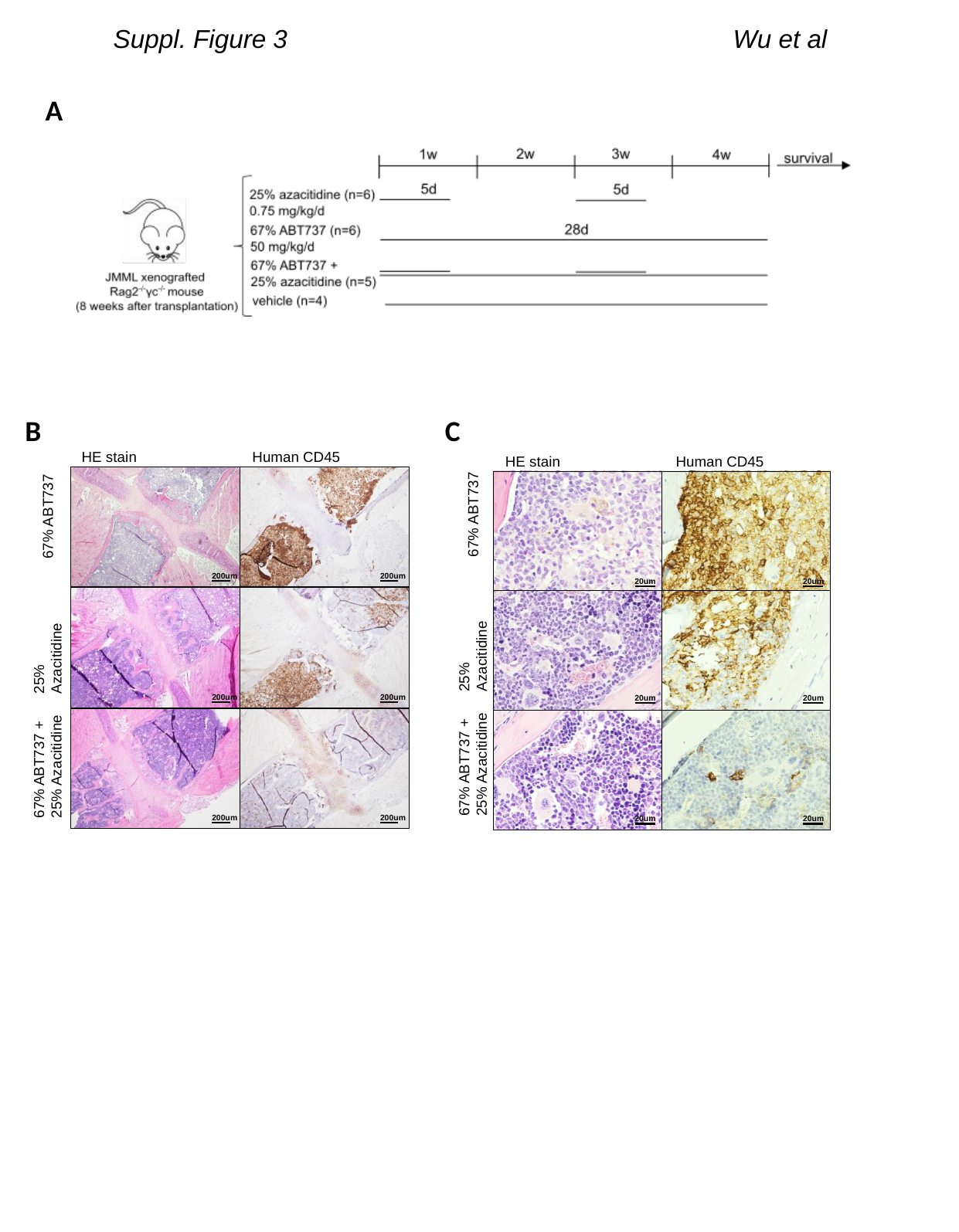

Suppl. Figure 3
Wu et al
A
B
C
HE stain
Human CD45
67% ABT737
200um
200um
25% Azacitidine
200um
200um
67% ABT737 +
25% Azacitidine
200um
200um
HE stain
Human CD45
67% ABT737
20um
20um
25% Azacitidine
20um
20um
67% ABT737 +
25% Azacitidine
20um
20um

## Slide 7
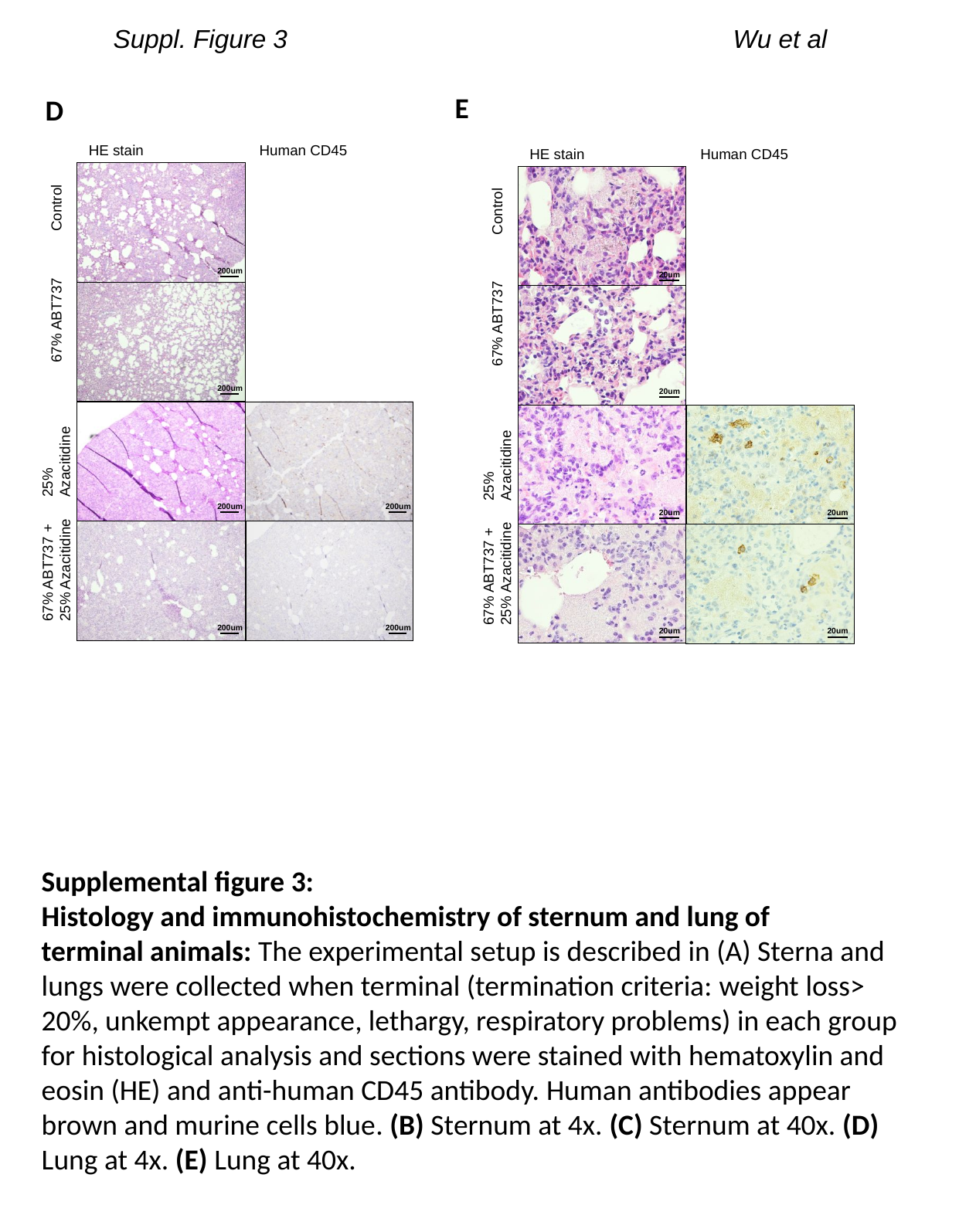

Suppl. Figure 3
Wu et al
E
D
HE stain
Human CD45
Control
200um
67% ABT737
200um
25% Azacitidine
200um
200um
67% ABT737 +
25% Azacitidine
200um
200um
HE stain
Human CD45
Control
20um
67% ABT737
20um
25% Azacitidine
20um
20um
67% ABT737 +
25% Azacitidine
20um
20um
Supplemental figure 3:
Histology and immunohistochemistry of sternum and lung of
terminal animals: The experimental setup is described in (A) Sterna and lungs were collected when terminal (termination criteria: weight loss> 20%, unkempt appearance, lethargy, respiratory problems) in each group for histological analysis and sections were stained with hematoxylin and eosin (HE) and anti-human CD45 antibody. Human antibodies appear brown and murine cells blue. (B) Sternum at 4x. (C) Sternum at 40x. (D) Lung at 4x. (E) Lung at 40x.

## Slide 8
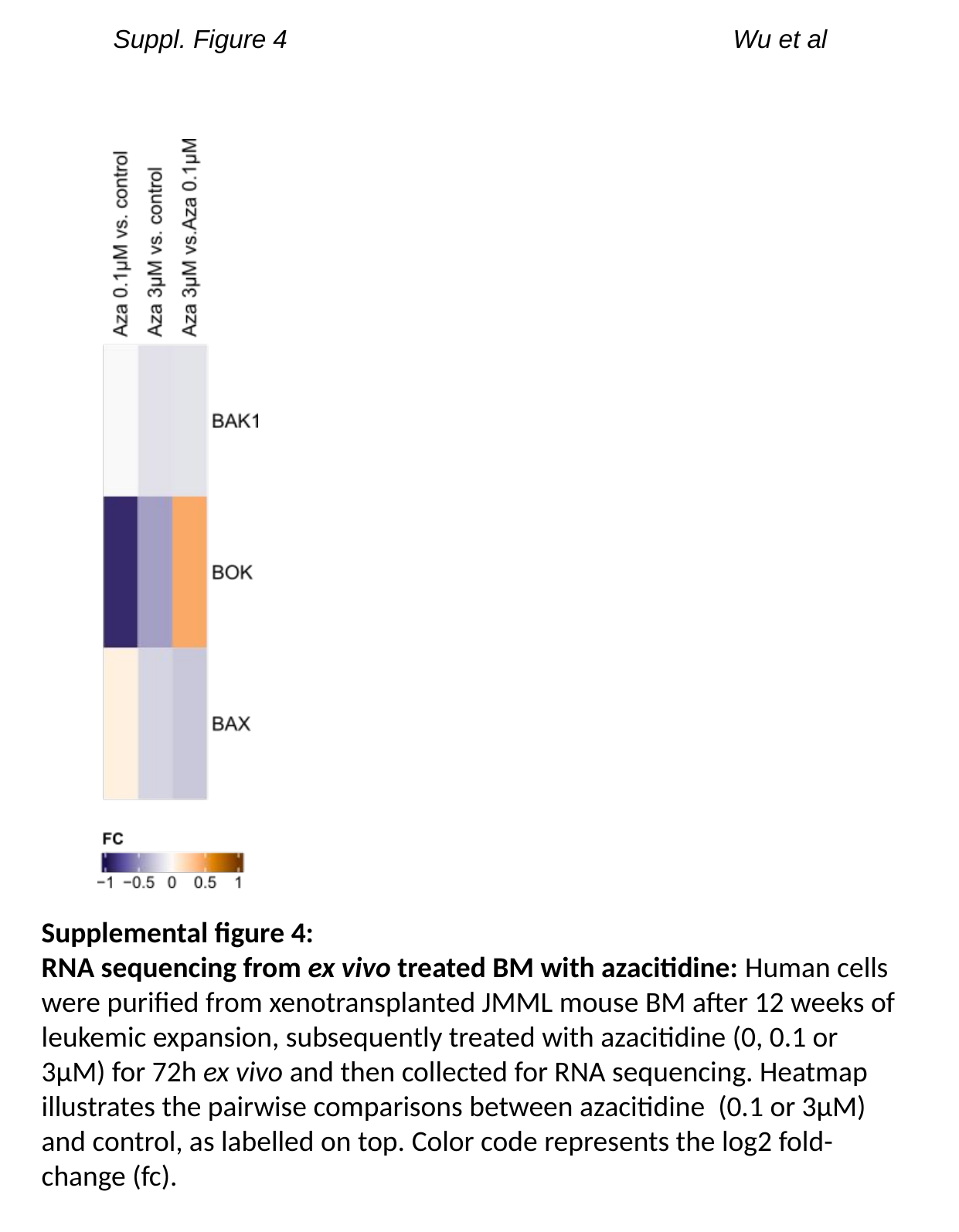

Suppl. Figure 4
Wu et al
Supplemental figure 4:
RNA sequencing from ex vivo treated BM with azacitidine: Human cells were purified from xenotransplanted JMML mouse BM after 12 weeks of leukemic expansion, subsequently treated with azacitidine (0, 0.1 or 3µM) for 72h ex vivo and then collected for RNA sequencing. Heatmap illustrates the pairwise comparisons between azacitidine (0.1 or 3µM) and control, as labelled on top. Color code represents the log2 fold-change (fc).
